# A decade of forensic odontology in India

**DOI:** 10.4103/0974-2948.71048

**Published:** 2010

**Authors:** Ashith B. Acharya

**Affiliations:** *Department of Forensic Odontology, S.D.M. College of Dental Sciences and Hospital, Sattur, Dharwad – 580009, Karnataka, India*. E-mail: *ashithacharya@hotmail.com*


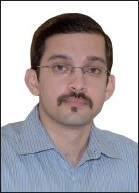


Interest in forensic odontology has been steadily rising over the past decade or so. The year 2000 was momentous for the specialty in India, when the Indian Association of Forensic Odontology (IAFO) was formed by a small group of enthusiastic dentists. The IAFO was subsequently registered in 2001 in Chennai, and yearly national conferences have been organized since 2002. It boasts of over 100 life members today, encompassing diverse dental specialties as well as private practitioners representing all corners of India.

Concurrently, a new breed of qualified forensic dentists returned to the country, having undertaken their postgraduation abroad. A few of them have managed to set up exclusive departments of forensic odontology in different parts of India. The inclusion of forensic odontology in the revised BDS curriculum in 2007 is also a landmark, enabling the subject’s exposure at an undergraduate level itself as part of oral pathology and oral medicine/radiology. Its inclusion as detailed chapters in numerous textbooks, most prominent among them being Shafer’s Textbook of Oral Pathology, has ensured that an overview of the subject is easily accessible to all dental students. The publication of this journal—the official publication of the IAFO—is also an integral and important component for increasing awareness of the specialty and encouraging research in it. Such dissemination of information and scholarly activity among dental students and dentists is vital for the subject’s growth and development—after all, it is dentists who can be expected to take the specialty forward. Proper knowledge among dentists ensures that the public, in general, and other stakeholders—particularly the police, forensic medical examiners and the judiciary—also recognize the specialty’s application and importance.

The routine use of forensic dentistry is, however, not yet a reality in India. While it is a foregone conclusion that every medico-legal case will entail a detailed forensic medical investigation, the same, however, is not true of forensic odontology. For this to fruition, sustained efforts are required by individual dentists interested and capable of contributing to the specialty, as well as organizations such as the IAFO. It is encouraging to note that this is ongoing. Private dental institutions are actively cooperating with state governments to ensure that public—private partnerships are established and the police routinely avail services offered in such institutions. Extensive interactions with the law enforcers (police), the judiciary (the judges) and the forensic fraternity (forensic medicine experts) at various forums, such as continual education programs, hands-on workshops and conferences, have increased the subject’s understanding, awareness and importance. The contribution of the media cannot be lost here—the fourth estate can single-handedly change the perception of the public on many matters; especially so, when it concerns the use of a science that ultimately aids in the preservation of law and order, and the furtherance of justice. That is the essence of forensic dentistry—contribution to the safety and well-being of our society.

